# Elongated styloid process evaluation on digital panoramic radiograph in a North Italian population

**DOI:** 10.4317/jced.53450

**Published:** 2017-03-01

**Authors:** Antonio Gracco, Alberto De Stefani, Giovanni Bruno, Paolo Balasso, Giulio Alessandri-Bonetti, Edoardo Stellini

**Affiliations:** 1 Prof, Department of Neuroscience, School of Dentistry, University of Padua, Padua, Italy; 2 DDS, Department of Neuroscience, School of Dentistry, University of Padua, Padua, Italy; 3 Dr, Department of Management and Engineering, University of Padova; 4 Prof, Department of Orthodontics, Alma Mater Studiorum University of Bologna, Bologna, Italy

## Abstract

**Background:**

The aim of this study is to evaluate the prevalence of elongated styloid process in digital panoramic radiographs in a North Italian population in relation to age, gender and side.

**Material and Methods:**

This study was performed as a retrospective analysis on digital panoramic radiographs of 600 (271 males and 329 females) Italian patients between 6 and 87 years old. The styloid process length were measured using the measuring tool of Sidexis Software. It was measured from the point where it left the temporal bone plate to its tip. Styloid processes measuring more than 30 mm were considered elongated. Chi-squared and Fisher tests were used and the test is considered significant if the *p*-value is lower or equal to 0.05.

**Results:**

Thirty-three per cent of the patients showed an elongated styloid process. Seventeen per cent were elongated on both right and left side, fifteen point nine per cent were elongated only in one side.

**Conclusions:**

The prevalence of elongated styloid process was high and a progressive increase of the elongation prevalence was found in older groups.

** Key words:**Elongated styloid process, Eagle’s syndrome, panoramic radiograph.

## Introduction

The styloid process is a cylindrical and sharp projection of the temporal bone, located in front of the stylomastoid foramen. It derives from Reichert’s cartilage of the second branchial arch so it is a part of the splancnocranium. It is connected to hyoid bone through the stylohyoid ligament. Styloid process is normally 20 to 30 mm in length. On the contrary, when it is longer than 30 mm it is defined elongated. Elongated styloid process is a common anatomical aspect in general population (1) and in most cases it is not associated to any clinical manifestation. When an elongated styloid process is associated to clinical symptoms of neck and orofacial pain, it characterizes Eagle’s syndrome. Patients show pain during head rotation, deglutition and mouth opening.

Morphological aspects of styloid process have been evaluated with different methods such as human dry skulls (2), digital panoramic radiographs (3,4), computed tomography (CT) (5,6) and finally on cone beam computed tomography (CBCT) (7,8). Even if CT and CBCT are more precise for measuring anatomical structures in three dimensions, digital panoramic radiographs are sufficiently accurate for the diagnosis of elongated styloid process. Moreover, digital panoramic radiographs are the primary resource for epidemiological studies due to their high distribution and easy interpretation (9,10).

Prevalence of elongated styloid process in digital panoramic radiographs has been studied in different populations resulting high variable (3,4,10,11). The high variability of this aspect among different races justifies an evaluation on Italian population since it has never been studied before.

The aim of this study is to evaluate the prevalence of elongated styloid process in digital panoramic radiographs in a North Italian population in relation to age, gender and side in order to compare it with the other population previously studied.

## Material and Methods

This study was performed as a retrospective analysis on digital panoramic radiographs of 600 (271 males and 329 females) Italian patients between 6 and 87 years old. It was decided to divide the sample size in four different age groups: patients younger than 18 years old, patients between 18 and 35 years old, between 36 and 53 and finally older than 54 years old.

The total sample size has been selected by appropriate statistical calculations by using R version 3.2.1. (R Core Team 2015) setting the power of the chi-squared test as 0.8. It resulted a sample that would have contained at least a number of patients with elongated Styloid Process equal or higher to 122.

This study was approved by the local ethical committee. The radiographs were selected from the Complex Operating Unit of Dentistry of Padua University Hospital database and they were all taken between 2014 and 2016. All the radiographs taken in our Dental Department are performed by the same operator and all the patients are positioned with their head oriented with the Frankfurt horizontal plane parallel to the floor. The radiographs were performed using a Sirona Ortophos XG (Sirona Dental Systems, Inc., USA). The radiographs setting were: a CCD sensor measuring 138 x 6.48 mm with 160 μm/pixel resolution, a tube voltage of 73 kV and a current of 15 mA, with 8.9 seconds of exposure time. The magnification factor reported by the manufacturer is 1.1.

Digital panoramic radiographs showing questionable styloid process, magnification errors, and superimposition of other anatomical structures were excluded from our evaluation.

**Figure 1:**
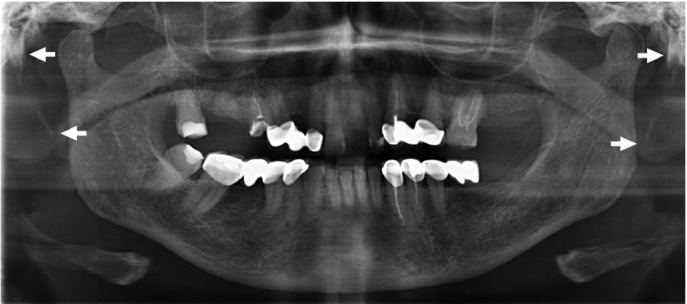
The styloid process length is measured from the point where it left the temporal bone plate to its tip.

The styloid process length were measured using the measuring tool of Sidexis Software (Sirona Dental Systems, Inc., USA). It was measured from the point where it left the temporal bone plate to its tip (Fig. 1). Styloid processes measuring more than 30 mm were considered elongated.

-Statistical analysis 

All data were entered into Excel 2010 (Microsoft, Remond, WA, USA). Statistical analyses were carried with the computing environment R version 3.2.1. that is used for estimating cross tabulation and the *p*-value of Chi-squared and Fisher tests. The test is considered significant if the *p*-value is lower or equal to 0.05. In the last table the Fisher’s test is performed instead of the Chi-squared test given that some frequencies were lower than 5%.

## Results

A total of 198 out 600 patients (33%) had radiographic images suggesting an elongated styloid process. 104 out 329 female (31.6%) and 94 out 271 male (34.6%) showed an elongated process. A *p*-value higher than 0.05 (0.425) detected no statistically significant dependence of gender in styloid process elongation even if male seemed to have a higher prevalence.

Regarding age, 6 out 100 patients (6%) under 18 years old, 33 out 108 (30.5%) patients between 18 and 35 years old, 55 out 132 (41.7%) patients between 36 and 53 years old and 104 out 260 (40%) patients over 54 years old showed an elongated styloid process. A statistically significant difference was found between the different groups with a *p*-value lower than 0.05 (0.0001) ( Table 1).

**Table 1:**
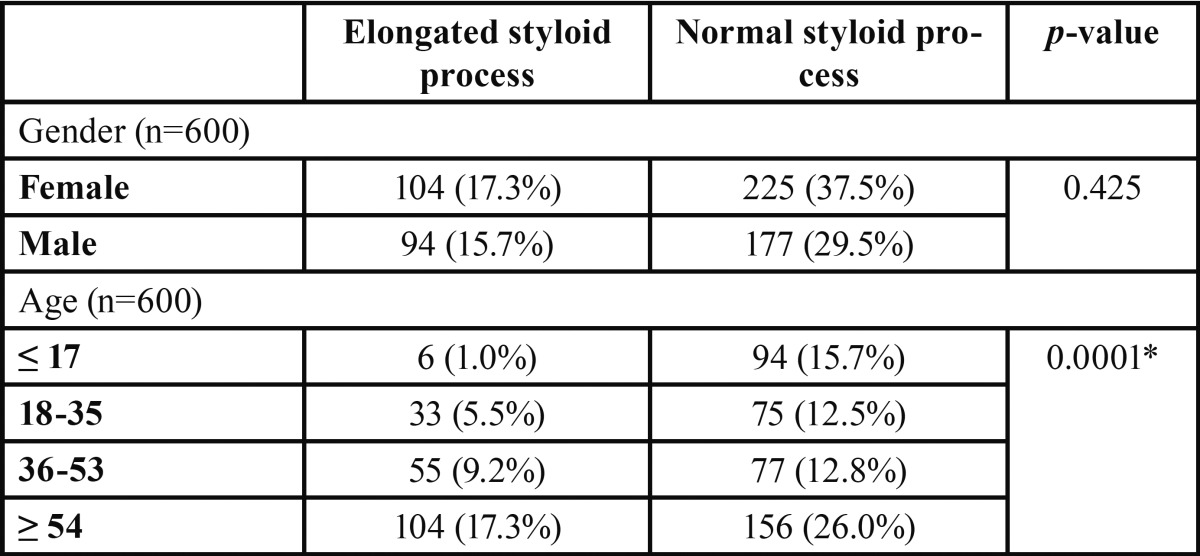
Absolute and relative frequencies of styloid processes according to gender and age and *p* values of Chi-square test.

Regarding lateral differences (bilateral and unilateral), this study showed that 102 styloid processes (17%) are elongated on both right and left side, while 96 styloid processes (15.9%) were elongated only in one side. No statistical significant was found between these 2 demographic information and affected side ( Table 2).

**Table 2:**
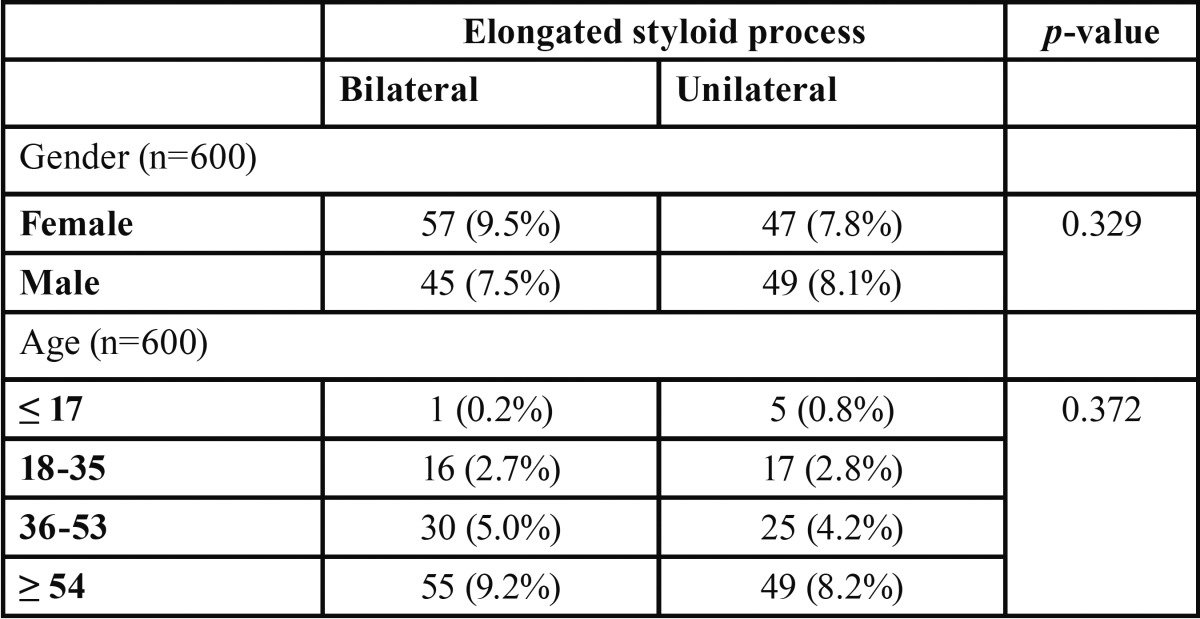
Absolute and relative frequencies of types of styloid processes according to gender and age.

## Discussion

Elongation of the styloid process is a frequent condition and its prevalence has been studied in various populations worldwide (3,4,10,11). According to international medical literature, in most cases it is not associated to any clinical symptom and it is reported that only a small percentage (about 5%) of patients presenting an elongated styloid process effectively shows a condition of orofacial and neck pain (12). In these cases it defines Eagle’s syndrome.

The elongation can be either bilateral or monolateral and its etiology is actually not well understood.

There are different signs and symptoms associated to Eagle’s syndrome: foreign body perception, throat pain, dysphagia, otologia, headache, face and neck pain especially during head rotation movement. These symptoms can be easily confused with other clinical conditions such as facial neuralgia, oral diseases, tooth diseases and temporomandibular disorders (13,14).

For this reason, clinical evaluation and radiographic records are essential tools to make a differential diagnosis. Several patients, in fact, require a dental or temporomandibular consult and dentists should to be able to discern Eagle’s syndrome from other diseases.

Eagle’s syndrome treatment is mainly surgical and according to anatomical particularities, the surgery can be performed with an intra-oral or extra-oral approach. Non-surgical treatment consists in non-steroidal anti-inflammatory medications and local infiltration with steroids or anaesthetic agents (15).

This is the first study that evaluates the prevalence of elongated styloid process in Italian population. Authors decide to use digital panoramic radiographs because they are generally the first exam made in a dental clinical practice, they are easy to interpret and they have a lower radiant dose than CT or CBCT (14,16,17). Moreover they have a lower cost than CT or CBCT.

In this study, the styloid process was measured from the point where it left the temporal bone plate to its tip using the method described by Ilgüy *et al.* (18).

The results of this study show a prevalence of 33% of elongated styloid process in a North Italian population.

This prevalence is different from those reported by other studies made on other populations: Vieira *et al.* (3) revealed a prevalence of 43.89% of elongated styloid process in Central Brazilian population, More and Asrani (19) revealed a prevalence of 19.4% in Indian population, Radfar *et al.* (11) revealed a prevalence of 22% in the US population. Our results are in according with previous panoramic radiographic studies reported in literature (13,17,20) which showed a prevalence of elongated styloid process ranging from 1.4 to 83.6%. This high variability can be ascribed either to real anatomical differences among different populations but also to different measurement criteria.

Regarding gender, a slightly higher prevalence of elongated styloid process was found in males than in females with a ratio of 1.1:1 but it resulted not statistically significant. This is a controversial point in medical literature: some authors found a higher prevalence of elongation in males (5,10,19), others found it in females (3,18) and finally someone did not find any difference related to gender (1,8).

In this study patients between 6 and 87 years old were evaluated and a statistically significant difference was found between the different age groups. We found a progressive increase of the elongation prevalence accordingly with the majority of the other studies (3,4,10,21). In further studies it would be interesting to establish if there is only a progressive increase of the number of patient showing an elongated styloid process (longer that 30mm) or even a progressive increase of the elongation itself with age. The data reported in this study confirm the idea that the progressive elongation is due to an age-related degeneration of the styloid ligament. Furthermore, in this study did not emerge a statistically significant difference on the side of the elongation. Half of the patients presented the elongation bilaterally and the other half presented it only in one side. Authors are not able to suggest a reason for this result since it is not already clear the etiology of the degeneration.

The aim of this study was to collect preliminary information on the prevalence of elongated styloid process in the Italian population since it had not been studied before and to compare it with similar studies on other races. Further studies with a bigger sample size are needed in order to evaluate the prevalence of elongated styloid process with a stricter sub-division of the age groups and to evaluate the increase in length of styloid process with advancing age.

Moreover, other studies using CBCT or CT are needed for a three-dimension evaluation of the styloid process and its relationship with other close anatomical structures.

In conclusion, the prevalence of elongated styloid process in North Italian population is 33%. The number of patients with elongated styloid process increase with the age. No statistically significant correlation is found between the presence of elongated styloid process and the gender and affected side (bilateral or unilateral).
